# Supplementary material for: LSD1 drives intestinal epithelial maturation and controls small intestinal immune cell composition independent of microbiota in a murine model
Source: Nat Commun. 2024 Apr 22;15:3412. doi: 10.1038/s41467-024-47815-2 (PMC11035651; doi:10.1038/s41467-024-47815-2)
Supplement: Supplementary file 1 — Supplementary Information [file 41467_2024_47815_MOESM1_ESM.pdf]

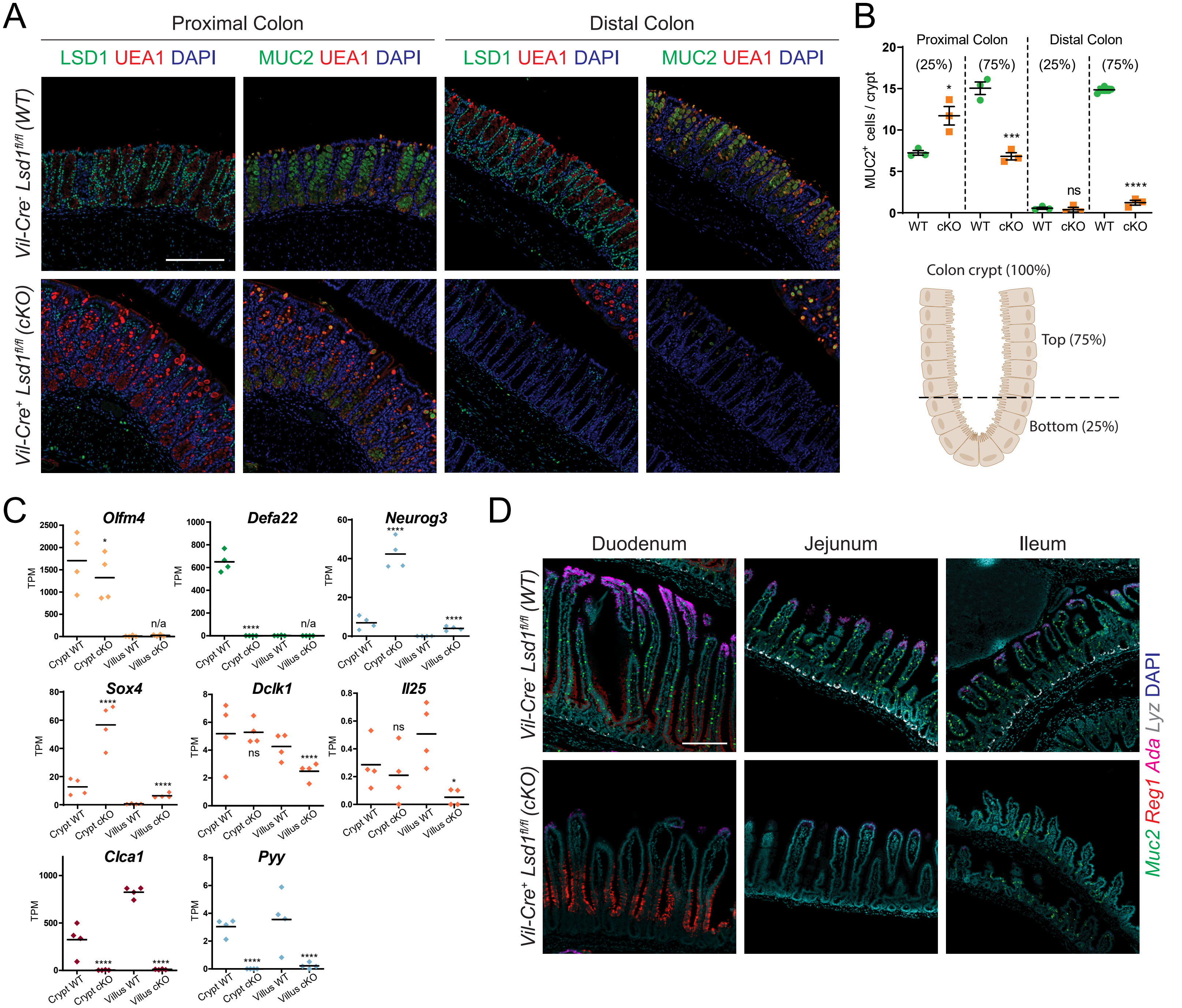

Fig. S1. LSD1 is required for the postnatal maturation of the intestinal epithelium. (A) Immunofluorescence staining of paraffin-embedded mouse colon tissue depicting complete loss of intestinal epithelial LSD1, and decreased goblet cells (MUC2+ UEA1+) in Lsd1 cKO mice, particularly in the distal portion. Scale bar: 200μm. **(B)** Quantification of goblet (MUC2+) cells across the colon (derived from Fig. S1A images). In order to depict the accumulation of immature/precursor goblet cells at the bottom of proximal colon crypts, crypts have been segmented according to their bottom 25% and top 75% portions. Data are presented as mean ± SEM in a scatter plot; n = 3 mice/genotype, (Two-tailed unpaired t-test). **(C)** Bulk RNA-seq of crypt and villus fractions derived from WT and cKO 2-month-old mice. Individual graphs show Transcripts per Million (TPM). Data are presented as mean & individual data points; n = 4 mice/genotype, (Differential expression analyzed using DESeq2's negative binomial generalized linear model, with Benjamini–Hochberg adjusted p-values). **(D)** Representative fluorescence in situ hybridization of key cellular markers for goblet (Muc2, green), Paneth (Lyz, white), villus-base (Reg1, red) and villus-tip (Ada, magenta) enterocytes. Scale bar: 200μm; n = 3 mice/genotype. Cell nuclei in all imaging are counterstained with DAPI.

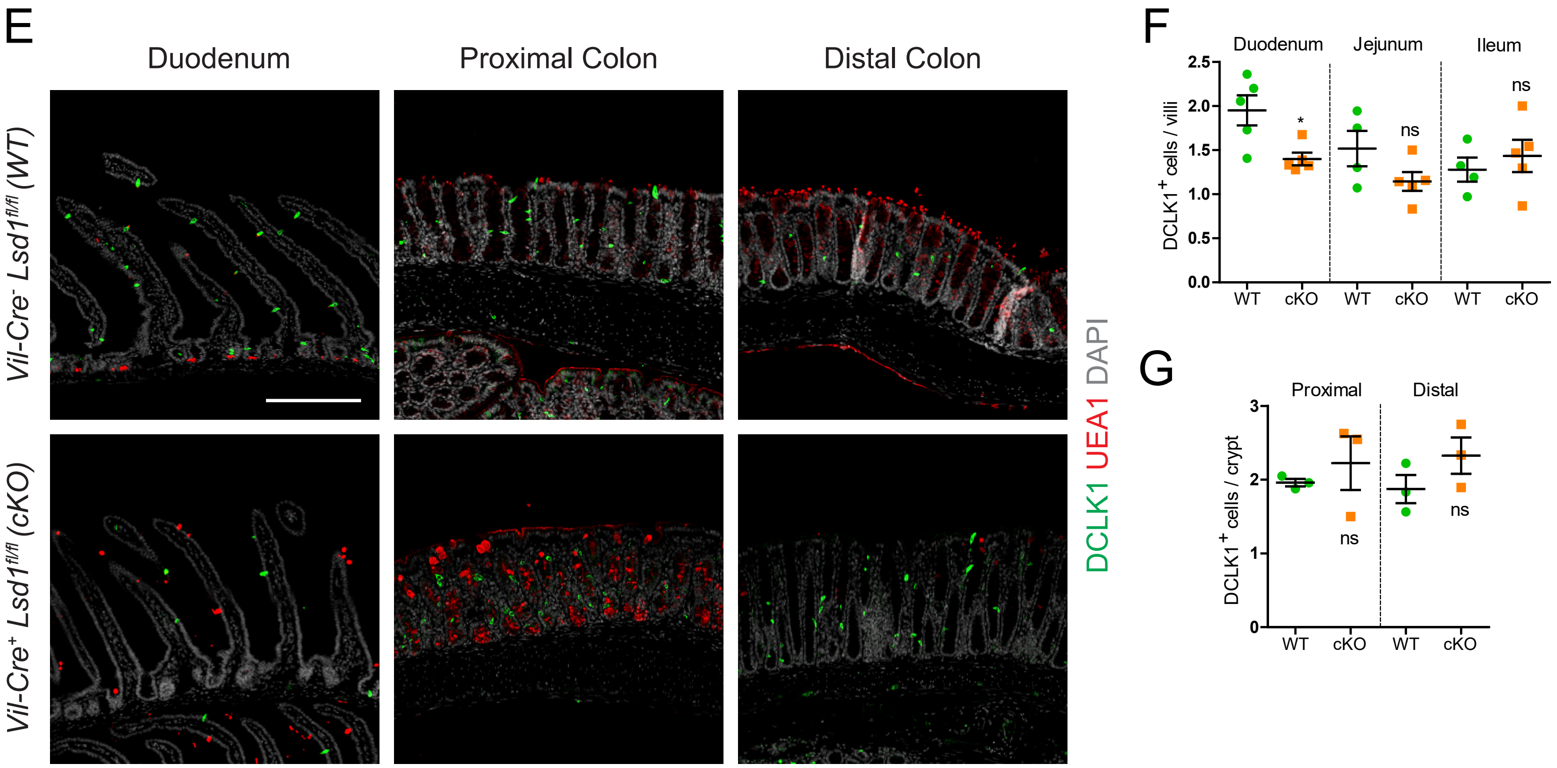

Fig. S1 (continued). LSD1 is required for the postnatal maturation of the intestinal epithelium. (E) Immunofluorescence staining of paraffin-embedded mouse small intestine and colon tissue depicting Tuft cells (DCLK1+) and Paneth and/or goblet cells (UEA1+) across the proximal-distal axis. Scale bar: 200µm. **(F)** Quantification of villus-residing Tuft (DCLK1+) cells across the small intestine (derived from Fig. S1E images). Data are presented as mean ± SEM in a scatter plot; n = 5 mice/genotype, (Two-tailed Mann-Whitney non-parametric test for Duodenum samples and Two-tailed unpaired t-test for normally distributed Jejunum and Ileum samples). **(G)** Quantification of Tuft (DCLK1+) cells across the colon (derived from Fig. S1E images). Data are presented as mean ± SEM in a scatter plot; n = 3 mice/genotype, (Two-tailed unpaired t-test).

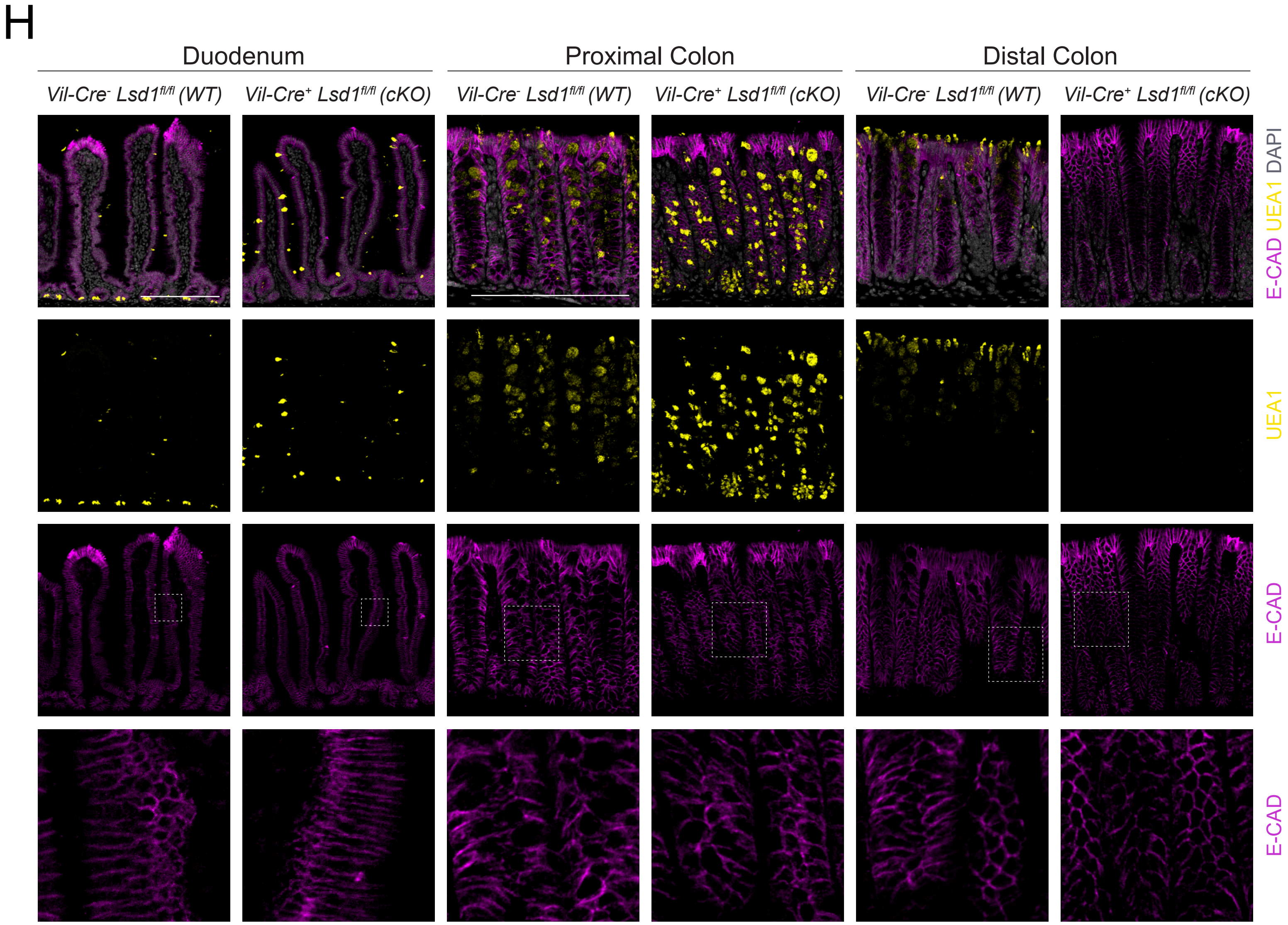

Fig. S1 (continued). LSD1 is required for the postnatal maturation of the intestinal epithelium. (H) Immunofluorescence staining of paraffin-embedded mouse small intestine and colon tissue depicting E-cadherin staining (magenta) as a proxy for adherens/tight junctions integrity. UEA1+ (yellow) is included as control to differentiate WT vs cKO tissue. Representative sections. All nuclei are counterstained with DAPI (grey). Scale bar: 200µm; ; n = 5 mice/genotype (small intestine) & n = 5 mice/genotype (colon).

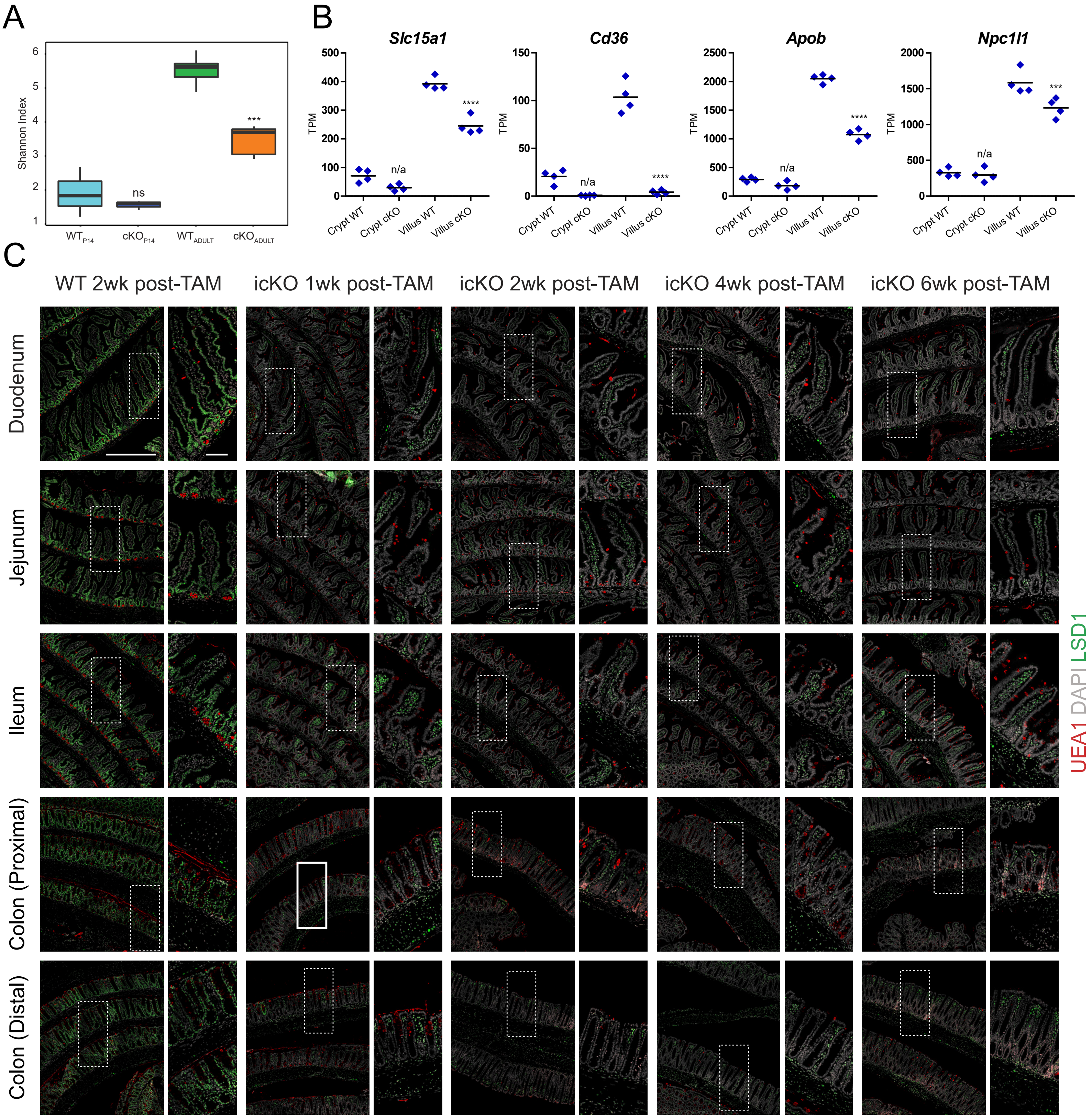

Fig. S2. Mature intestinal epithelium defines and maintains microbial composition. (A) Stool microbiota species diversity in adult (2-month-old) and P14 mice (WT and cKO); n = 5 adult mice/genotype & n = 3 P14 mice/genotype (Shannon Diversity Index). **(B)** Bulk RNA-seq of crypt and villus fractions derived from WT and cKO 2-month-old mice displaying C5 associated apolipoprotein transporters. Individual graphs show Transcripts per Million (TPM). Data are presented as mean & individual data points; n = 4 mice/genotype, (Differential expression analyzed using DESeq2's negative binomial generalized linear model, with Benjamini–Hochberg adjusted p-values). **(C)** Immunofluorescence staining of paraffin-embedded mouse intestinal tissue stained for LSD1 and UEA1 after tamoxifen treatment. Scale bar: 500µm, inset scale bar: 100µm; n = 5 mice/genotype from 2 independent experiments.

D

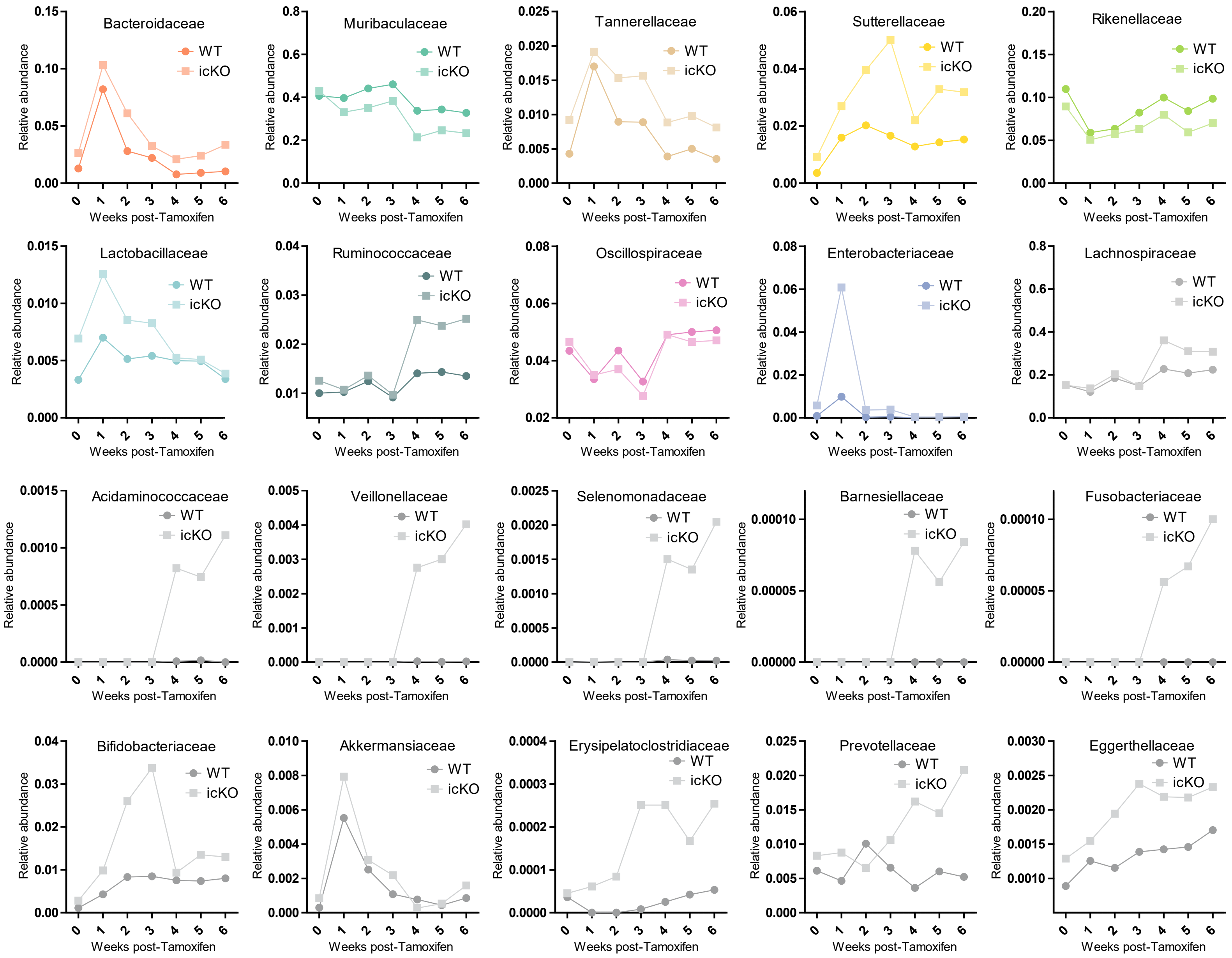

Fig. S2 (continued). Mature intestinal epithelium defines and maintains microbial composition. (D) Relative abundance representation of selected bacterial families before and after tamoxifen administration in WT and icKO litter and cagemates; n = 6 mice/genotype/timepoint.

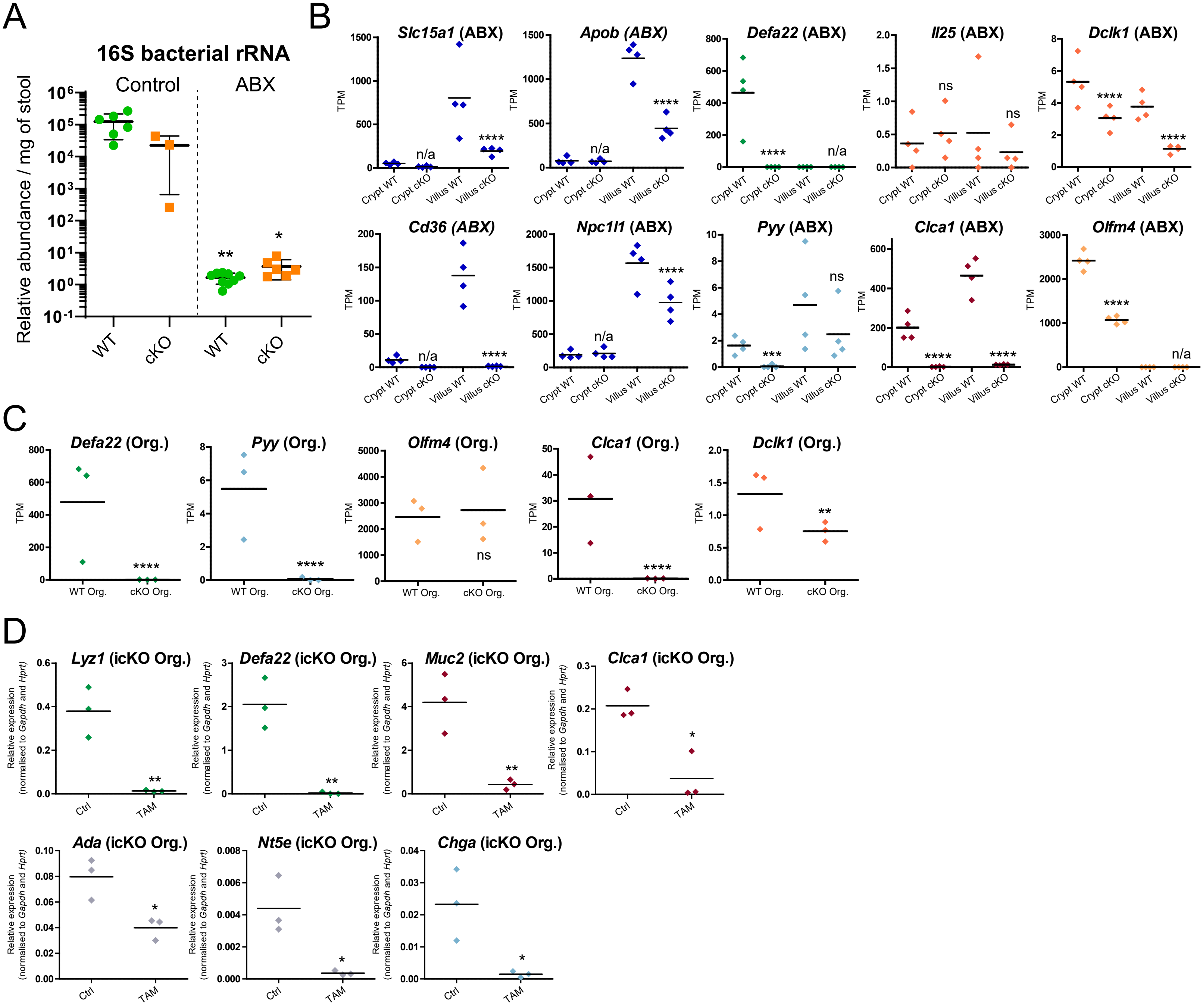

Fig. S3. LSD1 driven epithelial maturation is independent from the microbiota. (A) Relative bacterial 16S rRNA expression in stool samples derived from untreated (Control) or ABX-treated WT and cKO mice. Data are presented as mean ± SEM; n = 6 mice/WT Ctrl, n = 3 mice/ cKO Ctrl, n = 7 mice/Ctrl ABX, n = 6 mice/cKO ABX from 3 independent experiments, (One-tailed unpaired t-test, comparing Control vs ABX within the same genotype). **(B)** Bulk RNA-seq of crypt and villus fractions derived from WT (ABX) and cKO (ABX) 2-month-old mice. Individual graphs show Transcripts per Million (TPM). Data are presented as mean & individual data points; n = 4 mice/genotype, (Differential expression analyzed using DESeq2's negative binomial generalized linear model, with Benjamini–Hochberg adjusted p-values). **(C)** Bulk RNASeq of organoids derived from untreated WT and cKO mice. Individual graphs show Transcripts per Million (TPM). Data are presented as mean & individual data points; n = 3 mice/genotype, (Differential expression analyzed using DESeq2's negative binomial generalized linear model, with Benjamini–Hochberg adjusted p-values). **(D)** Gene expression profile derived from Vil-Cre^{ERT2+} Lsd1$^{fl/fl}$ (icKO) organoids treated *in vitro* with Tamoxifen. Relative gene expression assesed by qPCR. Data are presented as mean & individual data points; n = 3 mice/genotype/treatment from 2 indepen-dent experiments, (Two-tailed unpaired t-test).

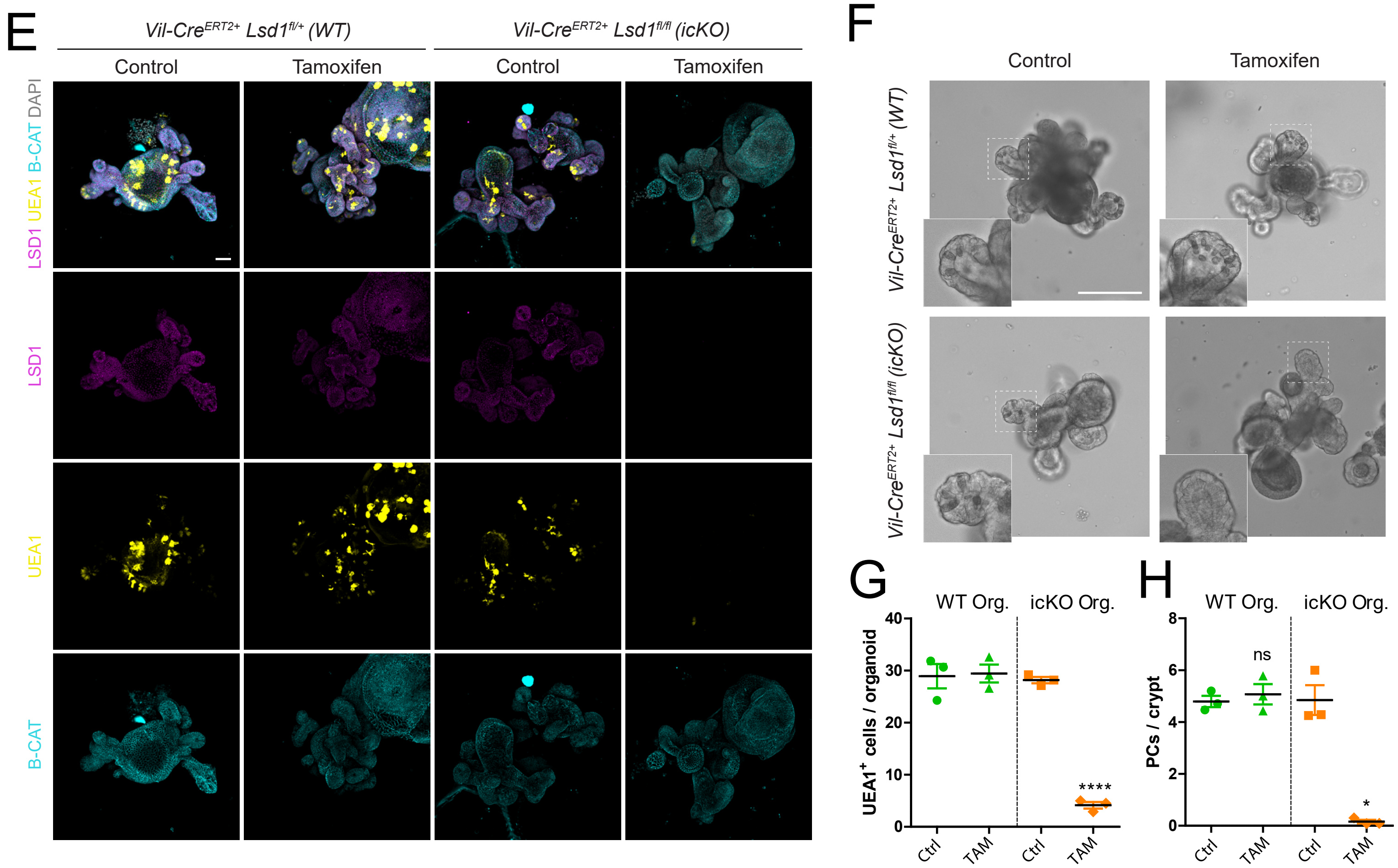

Fig. S3 (continued). LSD1 driven epithelial maturation is independent from the microbiota. (E) Organoids derived from WT and icKO mice were isolated and treated *in vitro* with Tamoxifen, passaged once and stained on day 4 post-passage. Control organoids are untreated. LSD1 (magenta), β-catenin (cyan) and goblet/Paneth cell (UEA1+, yellow) presence was determined by immunofluorescence; n = 3 mice/genotype/treatment. Scale bar: 50µm. **(F)** Brightfield images of Fig. S3D organoids pre-immunofluorescence staining. Note the absence of dark granulated cells (Paneth cells) in icKO organoids treated with Tamoxifen; n = 3 mice/genotype/treatment. Scale bar: 200µm. **(G)** Quantification of UEA1+ cells (goblet/Paneth cells) in organoids derived from WT or icKO mice untreated (Ctrl) or tamoxifen-treated (TAM) (derived from images such as shown in Fig. S3D. Data are presented as mean ± SEM; n = 3 mice/genotype/treatment from 2 independent experiments, (Two-tailed unpaired t-test). **(H)** Quantification of Paneth cells (PCs) per crypt in organoids derived from WT or icKO mice untreated (Ctrl) or tamoxifen-treated (TAM) (derived from images such as shown in Fig. S3E. Data are presented as mean ± SEM; n = 3 mice/genotype/treatment from 2 independent experiments, (One-tailed Mann-Whitney non-parametric test).

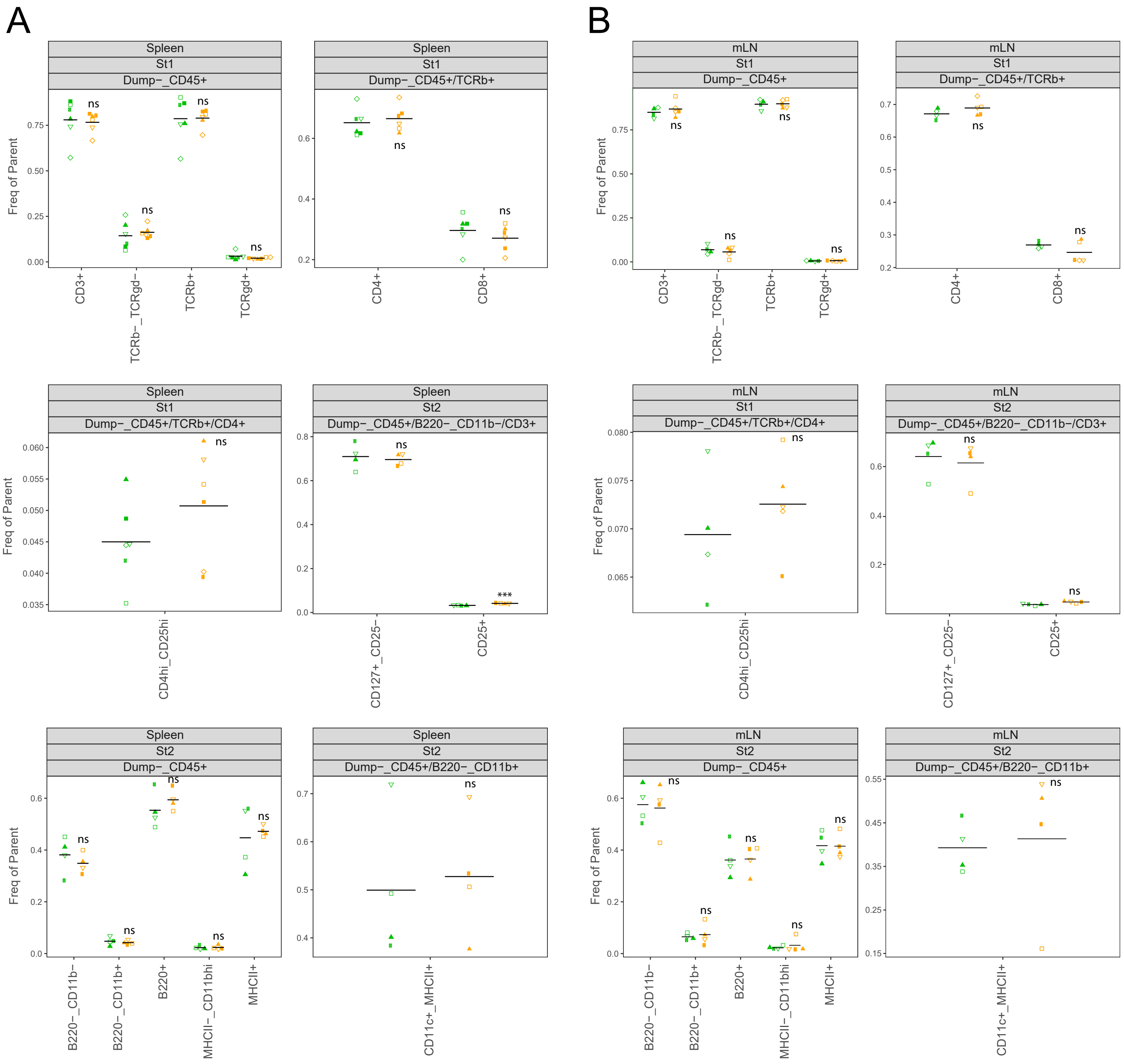

Fig. S4. LSD1-mediated intestinal epithelial maturation does not control systemic immune cell imbalance in spleen or mesenteric lymph nodes but directs local immune cell populations. (A & B) Flow cytometry data derived from spleen **(A)** or mLN **(B)** tissues showing frequency of B cells, T cell populations and myeloid cell populations. Green and orange data points correspond to WT and cKO mice respectively; Each data point represents and individual mouse, independent experiments were carried out for each WT and cKO pair, (Two-tailed unpaired t-test). St1 and St2 represent two different staining combinations. Parent gates are shown above each plot. Full gating hierarchy is shown in (S4E). Cells were gated on viable CD45+ cells. Ly6g+ granulocytes were excluded from the analysis. B220 was used as marker for B cells, and CD3, TCRb, TCRgd, CD4, CD8, CD25, CD127 were used to investigate T cell populations including CD4+ and CD8+ T helper cells, CD4hi CD25hi Tregs, and TCRb-TCRgd- "double-negative" T cells. CD11b, CD11c and MHCII were included to investigate myeloid cell populations including CD11c+ MHCII+ dendritic cells.

C

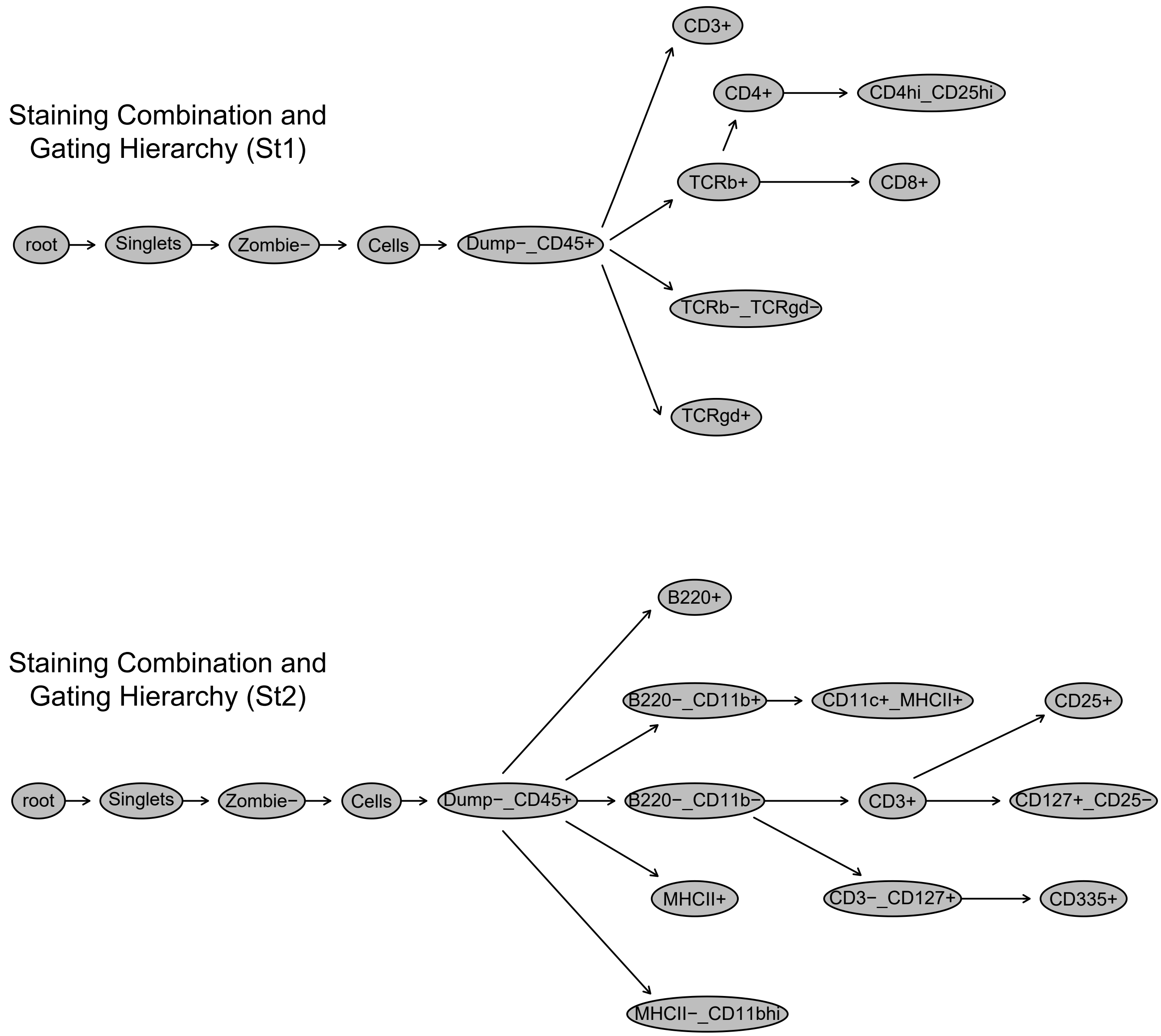

Fig. S4 (continued). LSD1-mediated intestinal epithelial maturation does not control systemic immune cell imbalance in spleen or mesenteric lymph nodes but directs local immune cell populations. (C) Complete gating hierarchy for analysis of immune cell populations by staining combinations St1 [CD3-BV605, CD8-BV785, CD25-AF488, TCRgd-PerCp-Cy5.5, TCRb-PE, CD4-APC, CD45-APC-Fire, Dump (CD326, CD19, CD11b, Ly6g, Ter119)-PE-Cy7] and St2 [CD335-BV421, CD3-BV605, CD127-BV711, CD25-BV785, MHCII-AF488, CD11c-PerCp-Cy5.5, B220-PE, CD11b-AF647, CD45-APC-Fire, Dump(CD326, Ly6g, Ter119)-PE-Cy7]. These staining combinations are referred to in Fig. S4A.

D

Gating Strategy (St1)

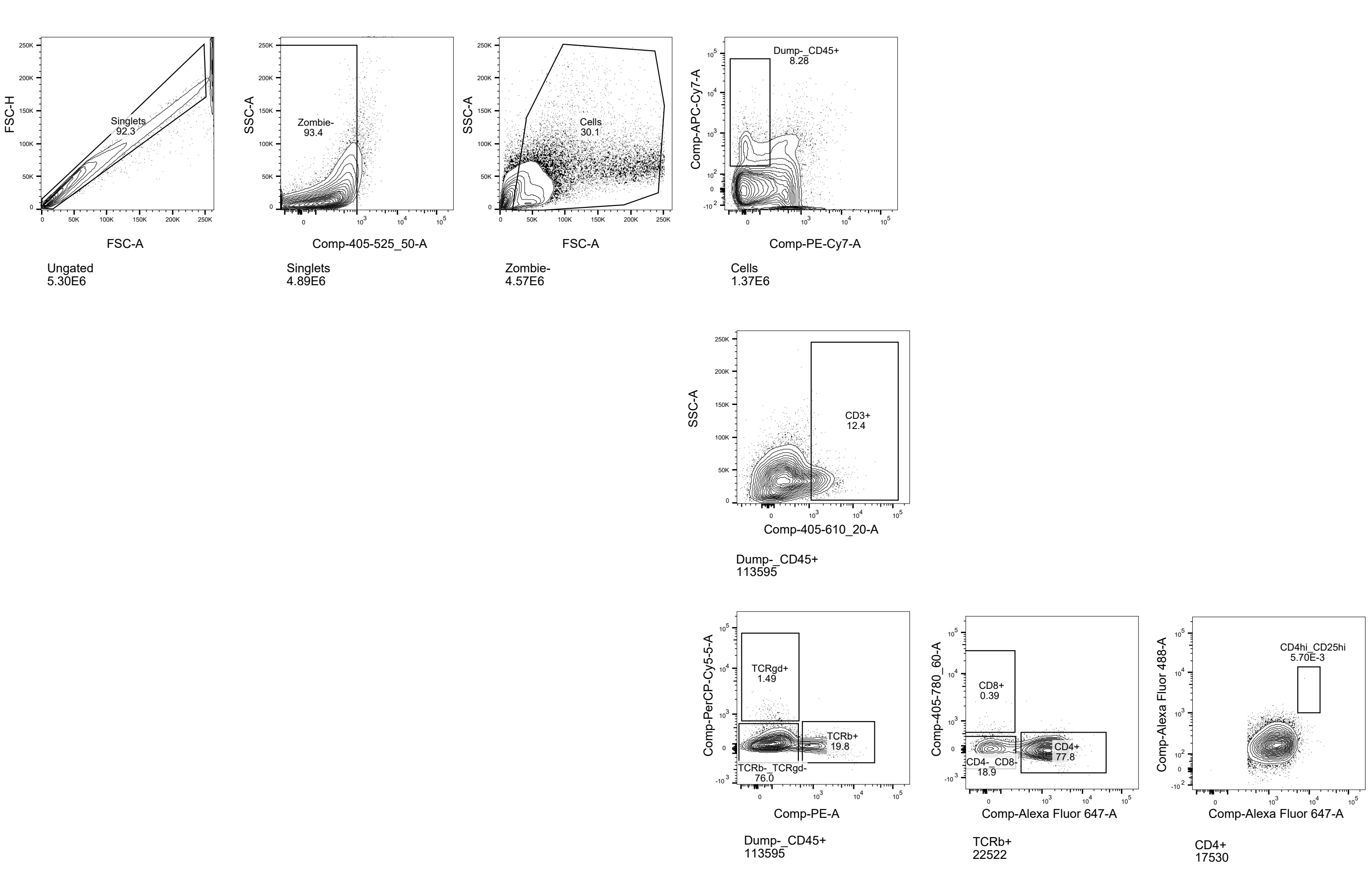

Fig. S4 (continued). LSD1-mediated intestinal epithelial maturation does not control systemic immune cell imbalance in spleen or mesenteric lymph nodes but directs local immune cell populations. (D) Complete gating strategy for analysis of immune cell populations by staining combination St1 [CD3-BV605, CD8-BV785, CD25-AF488, TCRgd-PerCp-Cy5.5, TCRb-PE, CD4-APC, CD45-APC-Fire, Dump (CD326, CD19, CD11b, Ly6g, Ter119)-PE-Cy7]. Gating strategy used in in Fig. S4A.

E

Gating Strategy (St2)

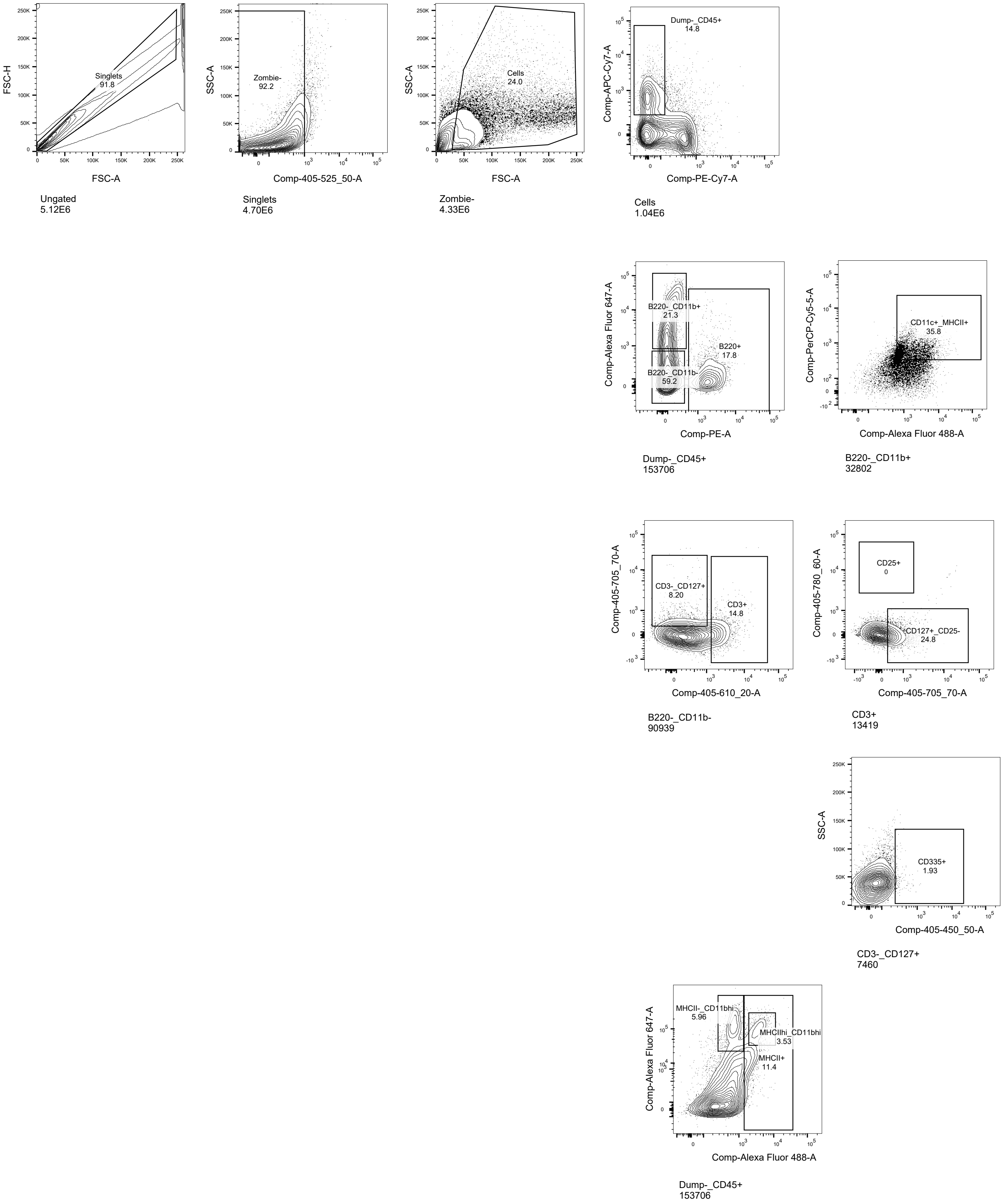

Fig. S4 (continued). LSD1-mediated intestinal epithelial maturation does not control systemic immune cell imbalance in spleen or mesenteric lymph nodes but directs local immune cell populations. (E) Complete gating strategy for analysis of immune cell populations by staining combination St2 [CD335-BV421, CD3-BV605, CD127-BV711, CD25-BV785, MHCII-AF488, CD11c- PerCp-Cy5.5, B220-PE, CD11b-AF647, CD45-APC-Fire, Dump(CD326, Ly6g, Ter119)-PE-Cy7]. Gating strategy used in in Fig. S4A and Fig. 6D.

F

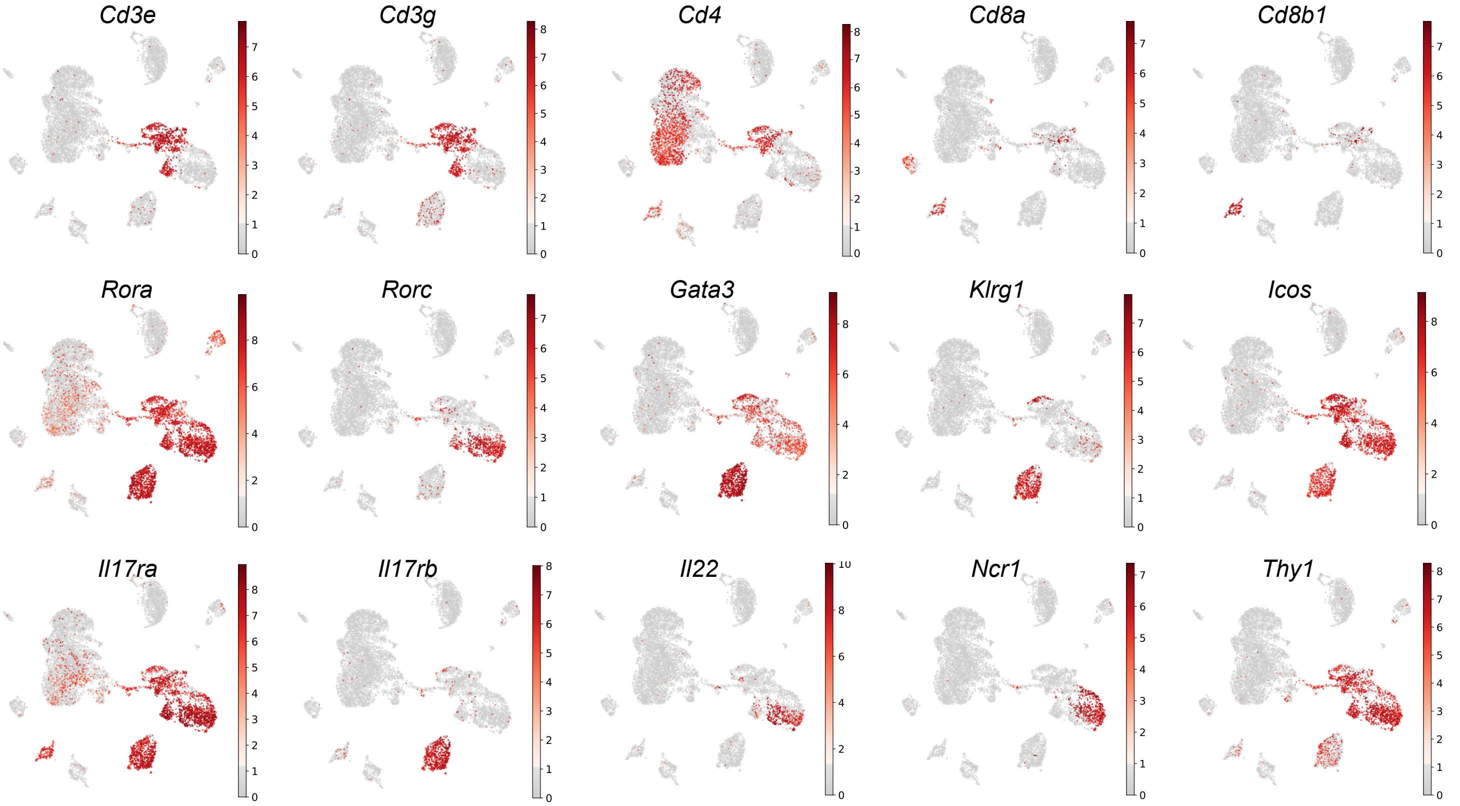

Fig. S4 (continued). LSD1-mediated intestinal epithelial maturation does not control systemic immune cell imbalance in spleen or mesen-teric lymph nodes but directs local immune cell populations. (F) UMAP of lamina propria CD45+-derived cells depicting individual gene expression across all experimental conditions merged (untreated WT, untreated cKO, ABX WT and ABX cKO). Grey to red heatmap scale shows log(Counts Per Million+1) or log(CPM+1).

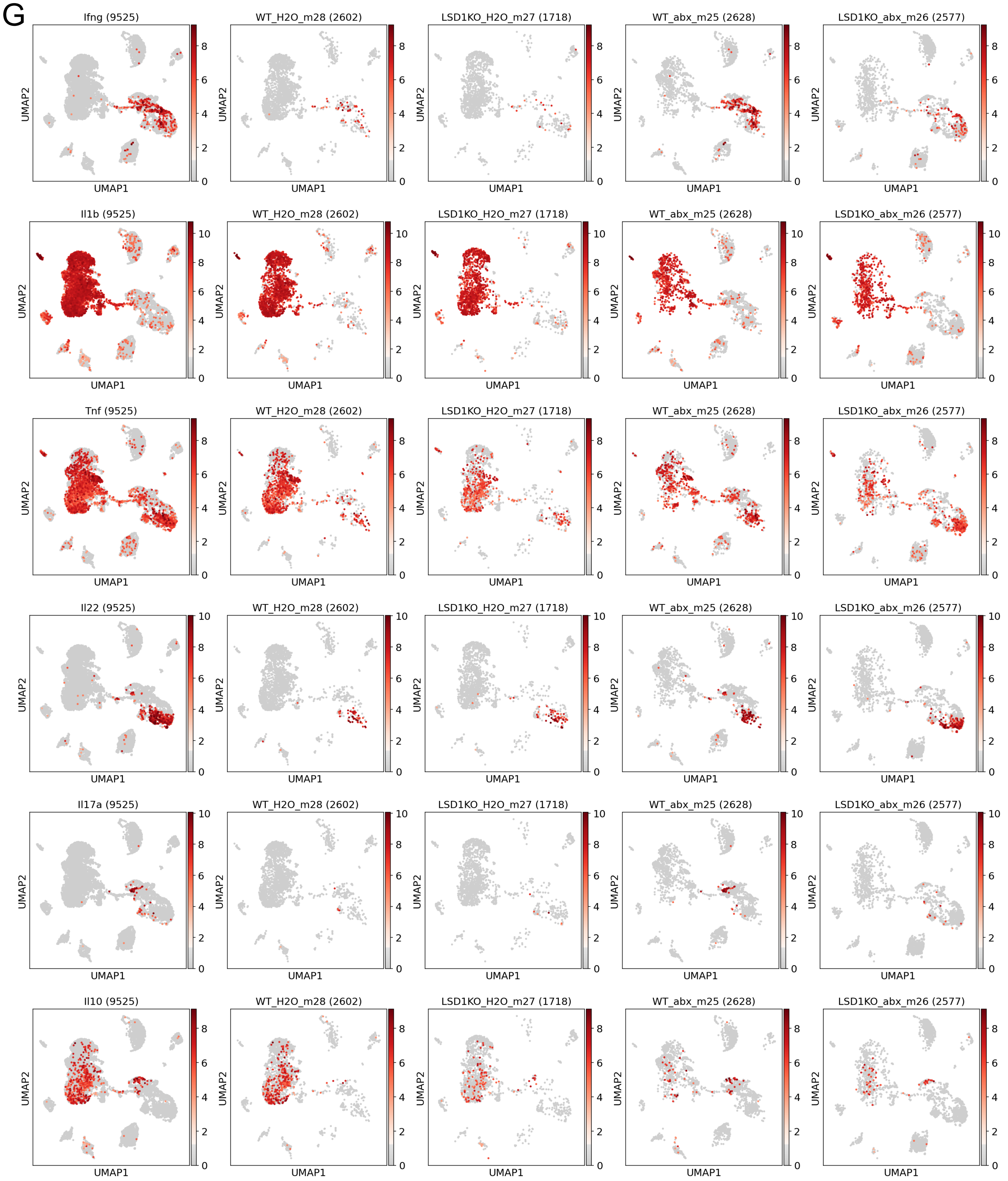

Fig. S4 (continued). LSD1-mediated intestinal epithelial maturation does not control systemic immune cell imbalance in spleen or mesen-teric lymph nodes but directs local immune cell populations. (G) UMAP of lamina propria CD45+-derived cells showing individual gene expression across all experimental conditions, from left to right: all conditions merged, untreated WT, untreated cKO, ABX WT and ABX cKO. Number in between parentheses represents the number of sequenced cells that passed quality control, (9525) corresponds to all four conditions merged under one UMAP. Grey to red heatmap scale shows log(CPM+1).

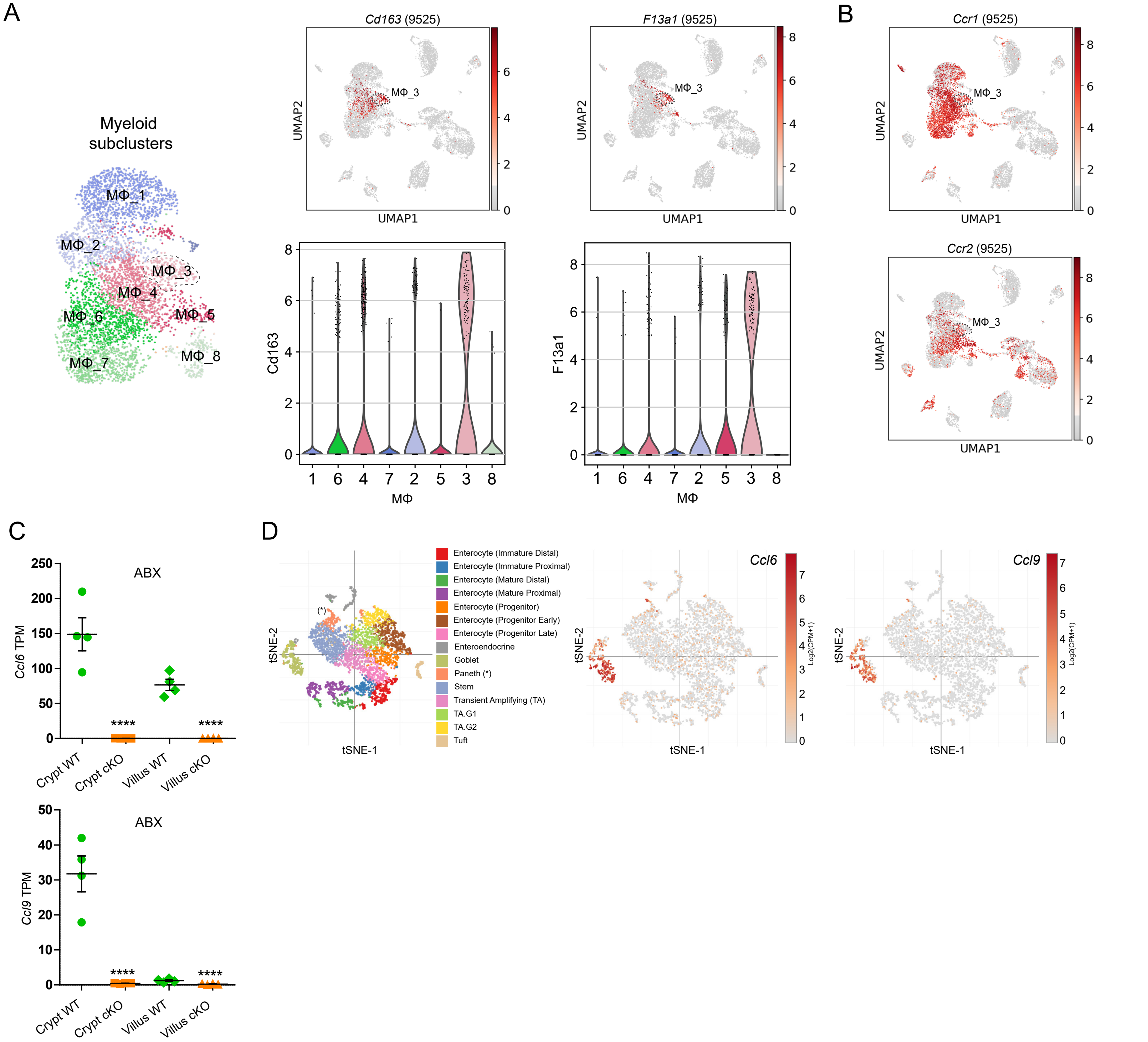

Fig. S5. LSD1-mediated intestinal epithelial maturation controls intestinal plasma cell and macrophage homeostasis. (A & B) UMAP of lamina propria CD45+-derived cells showing Ccr1, Ccr2, Cd163 and F13a1 gene expression across all experimental conditions. Number in between parentheses represents the number of sequenced cells that passed quality control, (9525) corresponds to all four conditions merged under one UMAP. Grey to red heatmap scale shows log(CPM+1). Violin plots in (A) show expression of indicated gene in each indicated myeloid subcluster (MΦ). Y-axis shows log(normalized count + 1) where count is normalized to the total number of reads per cell. **(C)** Bulk RNA-seq of crypt and villus fractions derived from WT and cKO ABX-treated 2-month-old mice. Individual graphs show Transcripts per Million (TPM). Data are presented as mean ± SEM; n = 4 mice/genotype, (Differential expression analyzed using DESeq2's negative binomial generalized linear model, with Benjamini–Hochberg adjusted p-values). **(D)** UMAP plot of cell types as determined with scRNA-seq from cells derived from the small intestinal epithelium of mice. Each cell type class is represented as a cluster of points in a unique color (left panel). UMAP showing Ccl6, and Ccl9 gene expression across cell types (middle and right panels). Grey to red heatmap scale shows log(CPM+1). Data derived from[29].

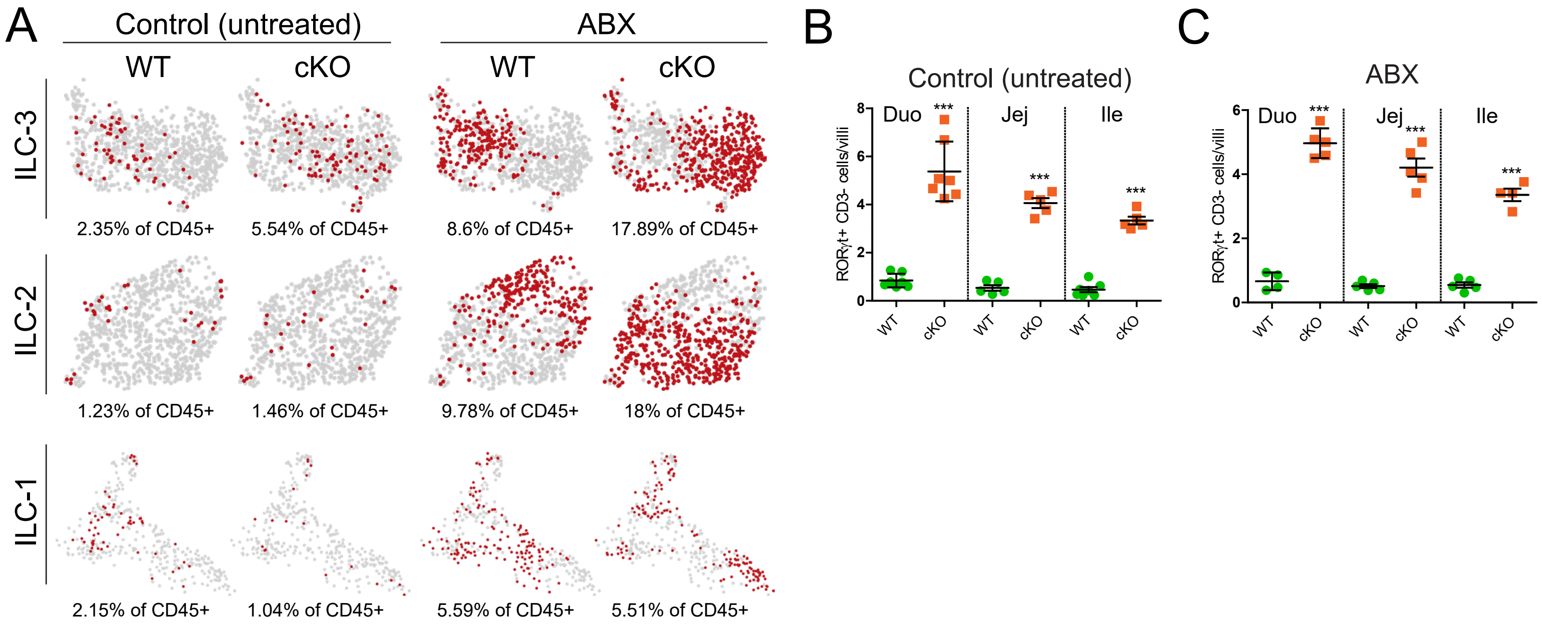

Fig. S6. LSD1-mediated intestinal epithelial maturation is required for the establishment and maintenance of ILC2s and ILC3s in a microbiota-independent manner. (A) UMAP clustering of lamina propria ILCs across all experimental conditions. Red dots correspond to the number of cells detected under each condition. Grey dots represent the sum of all detected ILCs across conditions. **(B)** Quantification of ILC3s in the lamina propria of untreated mice across the small intestine. Data are presented as mean ± SEM; n = 7 mice/genotype (duodenum) and 5 mice/genotype (jejunum & ileum) from 2 independent experiments, (Two-tailed Mann-Whitney non-parametric test for Duodenum samples and Two-tailed unpaired t-test for normally distributed Jejunum and Ileum samples). **(C)** Quantification of ILC3s in the lamina propria of ABX-treated mice across the small intestine. Data are presented as mean ± SEM; n = 5 mice/genotype from 2 independent experiments, (Two-tailed unpaired t-test).

D

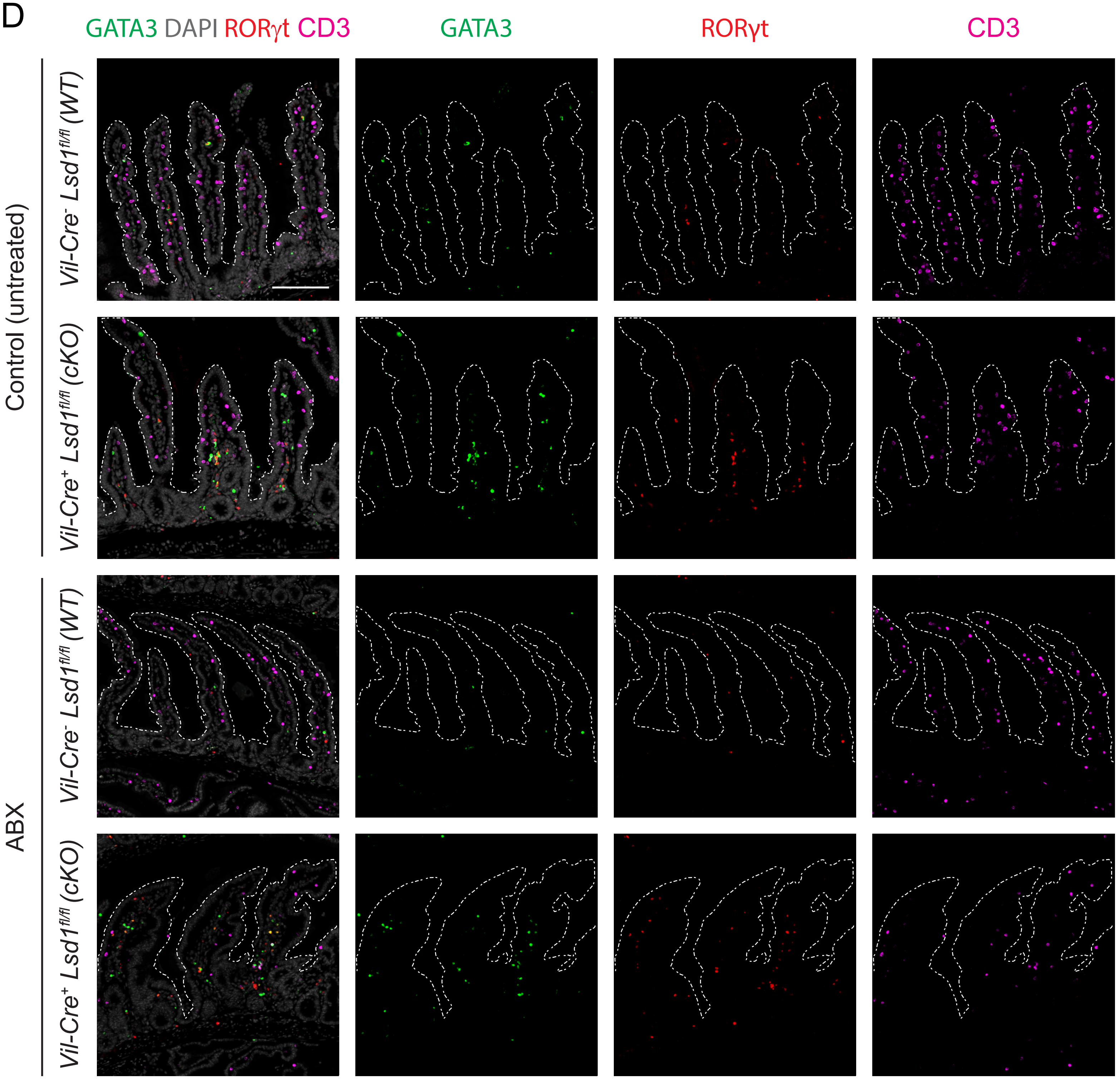

Fig. S6 (continued). LSD1-mediated intestinal epithelial maturation is required for the establishment and maintenance of ILC2s and ILC3s in a microbiota-independent manner. (D) Split channel immunofluorescence of mouse duodenums derived from Fig. 6A. ILC2s are defined as CD3- RORγt- GATA3+ while ILC3s are CD3- RORγt+. CD3 (magenta), RORγt (red), GATA3 (green) and nuclei are counterstained with DAPI (grey). Villus structure is delimited by a discontinuous white line; Scale bar: 200µm.

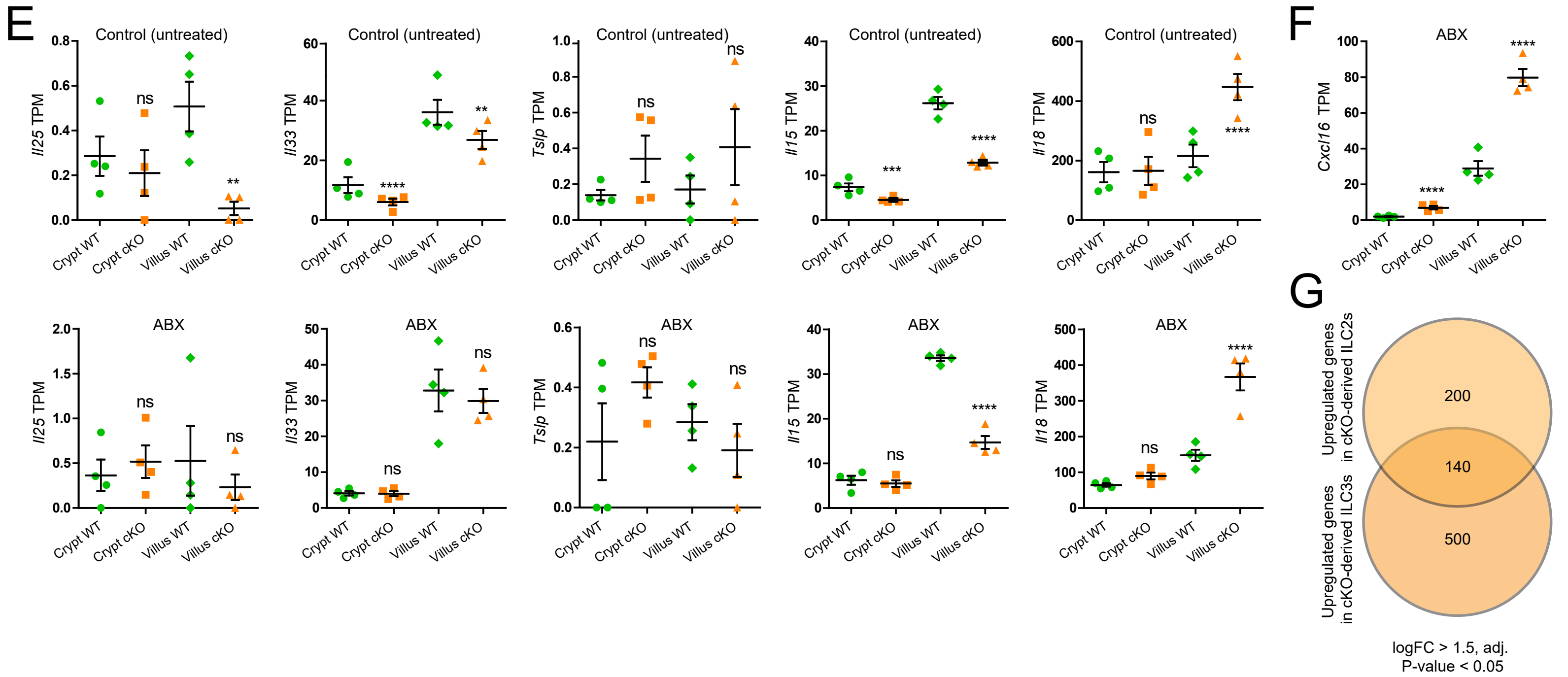

Fig. S6 (continued). LSD1-mediated intestinal epithelial maturation is required for the establishment and maintenance of ILC2s and ILC3s in a microbiota-independent manner. (E & F) Bulk RNA-seq of crypt and villus fractions derived from WT and cKO untreated or ABX-treated 2-month-old mice. Individual graphs show Transcripts per Million (TPM). Data are presented as mean ± SEM; n = 4 mice/genotype, (Benjamini–Hochberg adjusted p-value calculated with DESeq2). **(G)** Venn diagram showing overlapping upregulated genes found in both ILC2s and ILC3s derived from ABX treated mice.

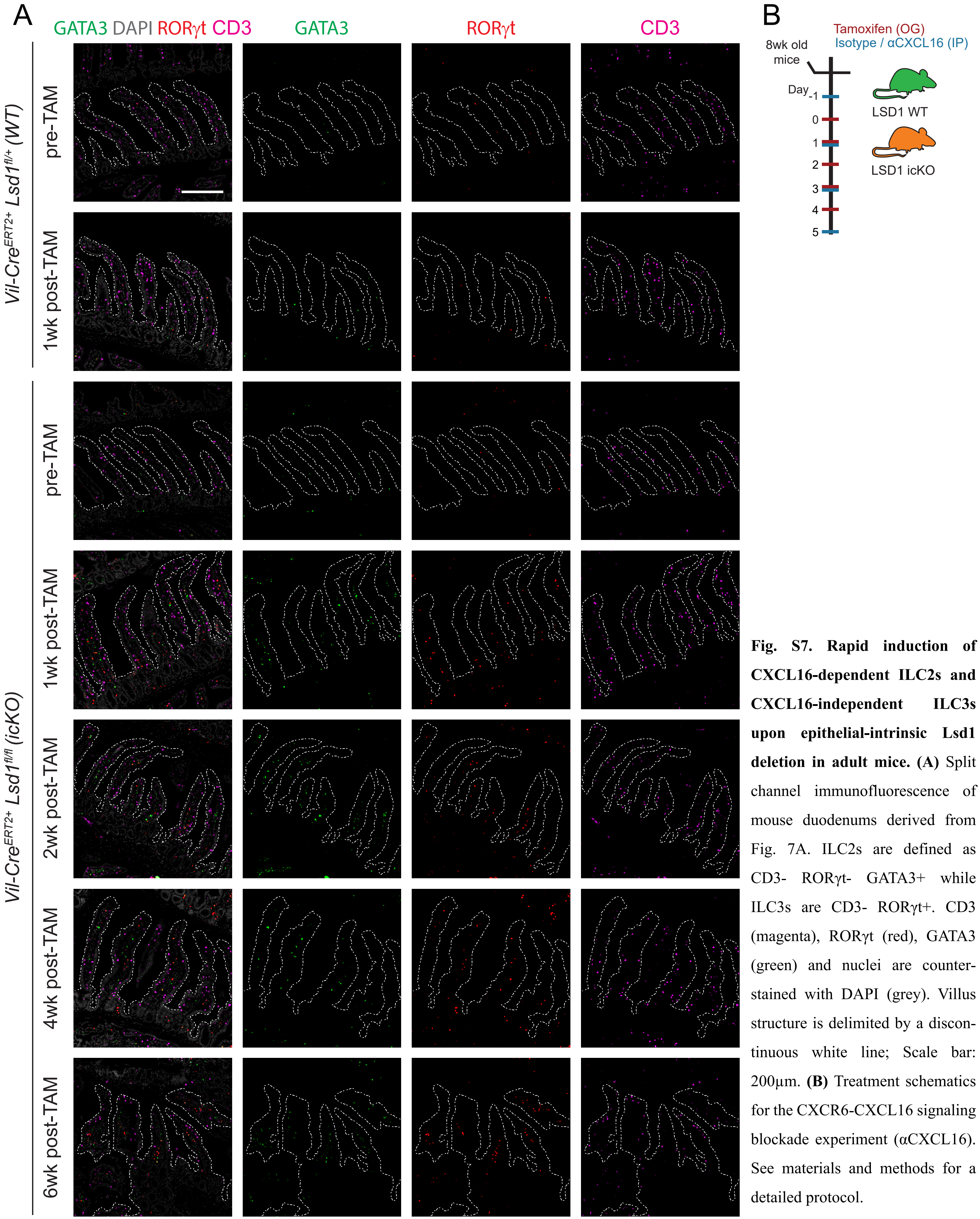

Fig. S7. Rapid induction of CXCL16-dependent ILC2s and CXCL16-independent ILC3s upon epithelial-intrinsic Lsd1 deletion in adult mice. (A) Split channel immunofluorescence of mouse duodenums derived from Fig. 7A. ILC2s are defined as CD3- RORγt- GATA3+ while ILC3s are CD3- RORγt+. CD3 (magenta), RORγt (red), GATA3 (green) and nuclei are counter-stained with DAPI (grey). Villus structure is delimited by a discontinuous white line; Scale bar: 200µm. **(B)** Treatment schematics for the CXCR6-CXCL16 signaling blockade experiment (αCXCL16). See materials and methods for a detailed protocol.

Gene Symbol	Gene Name	Primer Direction	Sequence
Lyz1	lysozyme 1	F	aagaatgcctgtgggatcaa
		R	ttgtatggctgcagtgatgtc
ChgA	chromogranin A	F	aacttcaagacctggctctcc
		R	ctcaaagctgctgtgttgct
Muc2	mucin 2	F	cacgagacccaggaagtacag
		R	gcaaagccactaactgcttgt
Defa22	defensin alpha 22	F	gatgaagagactaatactgaggagca
		R	cgttttctacaaaggcagatca
Clca1	chloride channel accessory 1	F	ctgggaggcaacacttttgg
		R	tcaggacaagggggagttga
Gapdh	glyceraldehyde-3-phosphate dehydrogenase	F	aggtcggtgtgaacggatttg
		R	tgtagaccatgtagttgaggtca
Hprt	hypoxanthine phosphoribosyltransferase	F	tcagtcaacgggggacataaa
		R	ggggctgtactgcttaaccag
Nt5e	5' nucleotidase, ecto	F	ggacatttgacctcgtccaat
		R	gggcactcgacacttggtg
Ada	adenosine deaminase	F	acccgcattcaacaaaccca
		R	agggcgatgcctctcttct

Table S1. Primer sequences for qPCR. Genes are all specific for *Mus musculus*.
