# Supplementary material for: LSD1 drives intestinal epithelial maturation and controls small intestinal immune cell composition independent of microbiota in a murine model
Source: Nat Commun. 2024 Apr 22;15:3412. doi: 10.1038/s41467-024-47815-2 (PMC11035651; doi:10.1038/s41467-024-47815-2)
Supplement: Supplementary file 3 — Description of Additional Supplementary Files [file 41467_2024_47815_MOESM3_ESM.pdf]

Description of Additional Supplementary Files

Supplementary Data 1. Collection of previously published genesets used in our GSEA pipelines. The geneset field corresponds to the identifier used in the figures. Source publication, gene count and gene names corresponding to each geneset are included.
