# Supplementary material for: LSD1 drives intestinal epithelial maturation and controls small intestinal immune cell composition independent of microbiota in a murine model
Source: Nat Commun. 2024 Apr 22;15:3412. doi: 10.1038/s41467-024-47815-2 (PMC11035651; doi:10.1038/s41467-024-47815-2)
Supplement: Supplementary file 5 — Reporting Summary [file 41467_2024_47815_MOESM5_ESM.pdf]

Corresponding author(s): Menno J. Oudhoff, Alberto Diez-Sanchez

Last updated by author(s): Mar 18, 2024

Reporting Summary

Nature Portfolio wishes to improve the reproducibility of the work that we publish. This form provides structure for consistency and transparency in reporting. For further information on Nature Portfolio policies, see our Editorial Policies and the Editorial Policy Checklist.

Statistics

For all statistical analyses, confirm that the following items are present in the figure legend, table legend, main text, or Methods section.

n/a	Confirmed	
☐	☒	The exact sample size (*n*) for each experimental group/condition, given as a discrete number and unit of measurement
☐	☒	A statement on whether measurements were taken from distinct samples or whether the same sample was measured repeatedly
☐	☒	The statistical test(s) used AND whether they are one- or two-sided<br>*Only common tests should be described solely by name; describe more complex techniques in the Methods section.*
☐	☒	A description of all covariates tested
☐	☒	A description of any assumptions or corrections, such as tests of normality and adjustment for multiple comparisons
☐	☒	A full description of the statistical parameters including central tendency (e.g. means) or other basic estimates (e.g. regression coefficient) AND variation (e.g. standard deviation) or associated estimates of uncertainty (e.g. confidence intervals)
☐	☒	For null hypothesis testing, the test statistic (e.g. *F*, *t*, *r*) with confidence intervals, effect sizes, degrees of freedom and *P* value noted<br>*Give P values as exact values whenever suitable.*
☒	☐	For Bayesian analysis, information on the choice of priors and Markov chain Monte Carlo settings
☒	☐	For hierarchical and complex designs, identification of the appropriate level for tests and full reporting of outcomes
☒	☐	Estimates of effect sizes (e.g. Cohen's *d*, Pearson's *r*), indicating how they were calculated

Our web collection on statistics for biologists contains articles on many of the points above.

Software and code

Policy information about availability of computer code

Data collection

Images were acquired with Zeiss Airyscan Software (Zen black V2.3) for tissue and organoid confocal images. EVOS2FL for brighfield imaging of organoids.
qPCR was performed in a StepOnePlus™ Real-Time PCR System (Applied Biosystems). Ct and melting curves were obtained with StepOnePlus™ Software v2.3 (Applied Biosystems).
Flow cytometry samples were analyzed using a BD LSR II flow cytometer (BD Biosciences) equipped with 405, 488, 561, 647 nm laser lines.

Data analysis

Bulk RNA-Seq analysis

Reads were aligned to the Mus musculus genome build mm10 using the STAR aligner[71]. The count of reads that aligned to each exon region of a gene in GENCODE annotation M18 of the mouse genome[72] was counted using featureCounts[73]. Genes with a total count less than 10 across all samples were filtered out. Differential expression analysis was done with the R package DESeq2, and volcano plots were plotted with the R package EnhancedVolcano[74]. PCA analysis was performed with the scikit-learn package with the function sklearn.decomposition.PCA[75]. GSEA analysis was run with the log2(fold change) calculated by DESeq2 as weights, 10,000 permutations, and otherwise default settings using the R package clusterProfiler[76]. GO term analysis was run using the function enrichGO in clusterProfiler. The R packages pheatmap and eulerr were used to make heatmaps and venndiagrams, respectively.

scRNA-Seq sequencing and analysis

scRNA-seq was performed using 10X genomics Chromium Next GEM Single Cell 3′ GEM, Library & Gel Bead Kit v3.1 and sequenced using two Illumina NS500HO flowcells with a 75 cycle kit. Reads were aligned to the genome and reads per gene was counted using the cellranger count function (v3.1.0) with the cellranger reference mm10 3.0.0[77]. Samples were then combined with cellranger aggr. Scanpy (v1.5.1)[78] was

used to quality filter the raw data with the following filters: min_genes_per_cell = 400, min_cells_per_gene = 3, max_counts_per_cell = 40000, max_percent_mt_per_cell = 20. Raw reads were converted to counts per million and log-transformed. Cell neighbours were computed with 10 neighbours and 40 PCA dimensions. Leiden algorithm was used with a resolution of 1 to split clusters and UMAP to display them. CIPR with the ImmGen dataset from mouse was used to initially identify the cell clusters79, and these were confirmed with cell type markers. Scanpy was used to make plots of cell clusters.

StringDB gene enrichment

Version 11.5 of STRING Database was used for gene enrichment and network graph display. Interaction sources to build up the network include: Textmining, experiments, databases, co-epxression, neighbourhood, gene fusion and co-occurrence. Only experimentally determined (magenta) and known interactions (cyan) from curated databases are depicted along interactions derived from textmining (green) and co-expression (black).

Microbial 16S Amplicon analysis

Amplicon sequence analysis was outsourced to Novogene UK. Paired-end reads was assigned to samples based on their unique barcodes and truncated by cutting off the barcode and primer sequences. After the barcode sequence and primer sequence were truncated, FLASH (v1.2.11, http://ccb.jhu.edu/software/FLASH/)80 was used to merge the reads to get raw tags. Then, fastp software was used to do quality control of raw tags, and high-quality clean tags were obtained. Finally, Vsearch software was used to blast clean tags to the database to detect the chimera and remove them, to obtain the final effective tags. For the effective tags, the DADA2 or deblur module in QIIME2 software was used to do denoise (DADA2 were used by default), and the sequences with less than 5 abundance were filtered out to obtain the final ASVs (Amplicon Sequence Variables) and feature table. Then, the Classify-sklearn moduler in QIIME2 software was used to compare ASVs with the database and to obtain the species annotation of each ASV. QIIME2 software was used to calculate alpha diversity indices including observed_ otus, shannon, simpson, chao1, goods_ coverage, dominance and pielou_e indices. The rarefaction curve and species accumulation boxplot were drawn. If there was grouping, the differences between groups of alpha diversity would be analyzed by default. For Beta Diversity analysis, the UniFrac distance was calculated by QIIME2 software, and the dimensionality reduction maps of PCA, PCoA and NMDS were drawn by R software. Among them, Ade4 and ggplot2 packages in R software were used to display PCA and PCoA. Then, Adonis and Anosim functions in QIIME2 software were used to analyze the significance of community structure differences among groups. Finally, LEfSe or R software was used to perform the species analysis of significant differences between groups. LEfSe analysis was performed by LEfSe software, and LDA score threshold was set to 4 by default. In MetaStat analysis, R software was used to test the differences between the two groups at the level of phylum, class, order, family, genus and species, and P value was obtained. The species with P value less than 0.05 were selected as the significant differences between the two groups; in T-test, R software was also used to analyze the significant differences of species at each taxonomic level.

Image Acquisition, Processing Software and Quantification

Images were acquired using Zen 2.3 Black and grey levels/maximum intensity projections were adjusted in Zen 2.3 Blue prior to .tiff export. Cell counts were manually quantified in an unbiased manner, blind to sample genotype, using sample numbers as identifiers. To quantify cells, we randomly selected complete and intact villi across the entire swiss roll. Based on availability, 25-30 morphologically complete villi were counted and averaged per biological replicate.

Flow Cytometry

For analysis, FlowJo software v10.6.2 and GNU R/Bioconductor v3.6.3/v3.10 packages flowCore, CytoML/flowWorkspace, ggcyto, flowViz were used.

Statistical Analysis

Unless stated otherwise in the methods section, statistical analysis and representation was carried out using Graphpad Prism V8.0.

Numbers in between correspond to references in the main text.

For manuscripts utilizing custom algorithms or software that are central to the research but not yet described in published literature, software must be made available to editors and reviewers. We strongly encourage code deposition in a community repository (e.g. GitHub). See the Nature Portfolio guidelines for submitting code & software for further information.

Data

Policy information about availability of data

All manuscripts must include a data availability statement. This statement should provide the following information, where applicable:

- Accession codes, unique identifiers, or web links for publicly available datasets
- A description of any restrictions on data availability
- For clinical datasets or third party data, please ensure that the statement adheres to our policy

We provide a Source Data file for all the presented graphs (Source_Data_1.xlsx). For gene expression graphs derived from bulk RNA-Seq experiments we supply and additional Source_Data_2_RNASeq_DiffExpression.xlsx. All sequencing data was uploaded via Annotare to the ArrayExpress repository at BioStudies EMBL-EBI (www.ebi.ac.uk/arrayexpress) under the following accession numbers. For bulk RNA-Seq-derived data: E-MTAB-10498 [https://www.ebi.ac.uk/biostudies/arrayexpress/studies/E-MTAB-10498] (Crypt and villi small intestinal epithelium from untreated WT and cKO mice), E-MTAB-10499 [https://www.ebi.ac.uk/biostudies/arrayexpress/studies/E-MTAB-10499] (Crypt and villi small intestinal epithelium from ABX-treated WT and cKO mice) and E-MTAB-10500 [https://www.ebi.ac.uk/biostudies/arrayexpress/studies/E-MTAB-10500] (Passage number 4, day 4 organoids derived from the duodenum of untreated WT and cKO mice). For scRNA-Seq-derived data: E-MTAB-10492 [https://www.ebi.ac.uk/biostudies/arrayexpress/studies/E-MTAB-10492]. For 16S rDNA Amplicon Metagenomic Sequencing of bacterial gDNA derived from mouse stool: E-MTAB-12489 [https://www.ebi.ac.uk/biostudies/arrayexpress/studies/E-MTAB-12489] (Adult vs Neonate) and E-MTAB-12626 [https://www.ebi.ac.uk/biostudies/arrayexpress/studies/E-MTAB-12626] (Timepoints before and after Lsd1 tamoxifen induced recombination in the intestinal epithelium). Raw imaging data and .fcs flow cytometry files can be shared on request.

The following datasets support the conclusions of the article and are cited in references:

29. Haber, A. L. et al. A single-cell survey of the small intestinal epithelium. Nature 2017 551:7680 551, 333–339 (2017).

30. Moor, A. E. et al. Spatial Reconstruction of Single Enterocytes Uncovers Broad Zonation along the Intestinal Villus Axis. Cell 175, 1156-1167.e15 (2018).

Genesets used for gene enrichment analysis are described in TableS1.genesets and under PMID: 26771021, 32917713 and 29144463.

Research involving human participants, their data, or biological material

Policy information about studies with human participants or human data. See also policy information about sex, gender (identity/presentation), and sexual orientation and race, ethnicity and racism.

Reporting on sex and gender	n/a
Reporting on race, ethnicity, or other socially relevant groupings	n/a
Population characteristics	n/a
Recruitment	n/a
Ethics oversight	n/a

Note that full information on the approval of the study protocol must also be provided in the manuscript.

Field-specific reporting

Please select the one below that is the best fit for your research. If you are not sure, read the appropriate sections before making your selection.

☒ Life sciences ☐ Behavioural & social sciences ☐ Ecological, evolutionary & environmental sciences

For a reference copy of the document with all sections, see nature.com/documents/nr-reporting-summary-flat.pdf

Life sciences study design

All studies must disclose on these points even when the disclosure is negative.

Sample size	Cohort size defined based on biological variation from pilot experiments & designed to give >80% power to detect 2-fold changes in expression patterns.<br>Sample size in organoids was determined based on biological variation in previous and related experiments.
Data exclusions	No data was excluded
Replication	We were able to replicate all results in replication experiments. Number of biological replicates are specified in each figure legend for each experiment.
Randomization	Mice were allocated randomly in each experimental group (genotype and or treatment)irrespective of sex. Littermates and/or age controls were used.
Blinding	All quantifications have been performed in an unbiased manner, blind to sample genotype, using sample (mouseID) numbers as identifiers.

Reporting for specific materials, systems and methods

We require information from authors about some types of materials, experimental systems and methods used in many studies. Here, indicate whether each material, system or method listed is relevant to your study. If you are not sure if a list item applies to your research, read the appropriate section before selecting a response.

Materials & experimental systems

n/a	Involved in the study	
☐	☒	Antibodies
☒	☐	Eukaryotic cell lines
☒	☐	Palaeontology and archaeology
☐	☒	Animals and other organisms
☒	☐	Clinical data
☒	☐	Dual use research of concern
☒	☐	Plants

Methods

n/a	Involved in the study	
☒	☐	ChIP-seq
☐	☒	Flow cytometry
☒	☐	MRI-based neuroimaging

Antibodies

Antibodies used

Immunofluorescence:

(LSD1, 1:200, Cell Signaling Technology, Cat. No. 2184S), (MUC2, 1:200, Santa Cruz Biotechnology, sc-15334), (LYZ, 1:200, Agilent DAKO, A0099), (β-CAT, 1:200, BD Biosciences, 610154), (CD3, 1:200, Novusbio, NB600-144155), (RORγt, 1:200, ThermoFisher, 12-6988-82), (GATA3, 1:200, Abcam, ab199428), (CD163, 1:200, Abcam, ab182422), (CD68, 1:200, BioRad, MCA1957T), (DCAMKL1/DCLK1, 1:200, Abcam, ab31704), (E-Cadherin (24E10), 1:200, Cell Signaling, 3195) and (IgA, 1:200, Novusbio, NB7503).

The following secondary antibodies from Invitrogen (Thermo Fisher Scientific) were used at a 1:500 dilution for immunostaining: (Goat anti Rabbit 488, A-11034), (Goat anti Mouse 555, A-21422), (Goat anti Rabbit 555, A-21428), (Goat anti Rat 555, A-21434), (Goat anti Mouse 647, A-21237), (Goat anti Rabbit 647, A-21245) and (Goat anti Rat 647, A-21247).

In vivo blockade experiment: Monoclonal rat IgG2A CXCL16 neutralizing antibody (R&D Systems, Clone #142417, MAB503) or monoclonal Rat IgG2A isotype control (R&D Systems, Clone #54447, MAB006)

Validation

We commonly use negative controls (no primary but secondary antibody or control isotypes) for all of our stainings/blocking experiments. All primary antibodies have been described/used before for the species and methods used as reported in their corresponding commercial website. See catalog numbers above for a detailed statement on validation on the manufacturer's website.

Animals and other research organisms

Policy information about studies involving animals; ARRIVE guidelines recommended for reporting animal research, and Sex and Gender in Research

Laboratory animals

C57BL/6 background LSD1KO (Villin-Cre Lsd1 fl/fl, cKO) and C57BL/6 background i-LSD1KO (Villin-CreERT2 Lsd1 fl/fl, icKO) strains were used (both male and females). Mice were housed in a SPF facility, with controlled temperature between 21 and 22 degrees Celsius and relative humidity between 45 and 60%. The animals are housed in a 12 hour dark/12 hour light cycle, with 1 hour of dusk/dawn. All mice used were between 8-14 weeks of age.

Wild animals

No wild animals were used in the study

Reporting on sex

Experimental groups contain a 50-50 male-female sex distribution since our observations are not affected by sex.

Field-collected samples

No field-collected samples were used in the study

Ethics oversight

Norwegian Food Safety Authority, FOTS protocol (FOTS ID 21275). Ethical committee was constituted by Ole Aamodt and Vera Klafstad Rodas.

Note that full information on the approval of the study protocol must also be provided in the manuscript.

Plants

Seed stocks

Report on the source of all seed stocks or other plant material used. If applicable, state the seed stock centre and catalogue number. If plant specimens were collected from the field, describe the collection location, date and sampling procedures.

Novel plant genotypes

Describe the methods by which all novel plant genotypes were produced. This includes those generated by transgenic approaches, gene editing, chemical/radiation-based mutagenesis and hybridization. For transgenic lines, describe the transformation method, the number of independent lines analyzed and the generation upon which experiments were performed. For gene-edited lines, describe the editor used, the endogenous sequence targeted for editing, the targeting guide RNA sequence (if applicable) and how the editor was applied.

Authentication

Describe any authentication procedures for each seed stock used or novel genotype generated. Describe any experiments used to assess the effect of a mutation and, where applicable, how potential secondary effects (e.g. second site T-DNA insertions, mosiacism, off-target gene editing) were examined.

Flow Cytometry

Plots

Confirm that:

☒ The axis labels state the marker and fluorochrome used (e.g. CD4-FITC).

☒ The axis scales are clearly visible. Include numbers along axes only for bottom left plot of group (a 'group' is an analysis of identical markers).

☒ All plots are contour plots with outliers or pseudocolor plots.

☒ A numerical value for number of cells or percentage (with statistics) is provided.

Methodology

Sample preparation	Single cells were stained with Zombie Aqua (Biolegend, 1:1,000 in PBS) for 15 min at room temperature (RT) for live-dead exclusion. Samples were incubated with two different panels (St1 and St2), consisting of antibody conjugates against: [St1: CD335-BV421, CD3-BV605, CD127-BV711, CD8-BV785, CD25-AF488, TCRgd-PerCp-Cy5.5, TCRb-PE, CD4-APC, CD45-APC-Fire, Dump (CD326, CD19, CD11b, Ly6g, Ter119)-PE-Cy7] and [St2: CD335-BV421, CD3-BV605, CD127-BV711, CD25-BV785, MHCII-AF488, CD11c- PerCp-Cy5.5, B220-PE, CD11b-AF647, CD45-APC-Fire, Dump(CD326, Ly6g, Ter119)-PE-Cy7] [all Biolegend, 1:200 in PBS + 2% fetal calf serum (FCS)] for 20 min at 4°C. Single fluorochrome stainings of cells and compensation particles (BD CompBead, Becton Dickinson) were included in each experiment.
Instrument	All the samples were analyzed using a BD LSR II flow cytometer (BD Biosciences) equipped with 405, 488, 561, 647 nm laser lines.
Software	For analysis, FlowJo software v10.6.2 and GNU R/Bioconductor v3.6.3/v3.10 packages flowCore, CytoML/flowWorkspace, ggcyto, flowViz were used.
Cell population abundance	Abundance is reported for all described cell populations
Gating strategy	A figure exemplifying the gating strategy is provided in the Supplementary Information

☒ Tick this box to confirm that a figure exemplifying the gating strategy is provided in the Supplementary Information.
